# Relationship Between Salt Intake and Cardiovascular Disease

**DOI:** 10.1111/jch.70078

**Published:** 2025-06-23

**Authors:** Fuzhou Han, Wenqiang Li, Ning Duan, Xinlong Hu, Nan Yao, Guoyong Yu, Jun Qu

**Affiliations:** ^1^ Department of General Surgery Aerospace Center Hospital Beijing China; ^2^ Department of Nephrology Beijing University of Chinese Medicine Affiliated Dongzhimen Hospital Beijing China

**Keywords:** cardiovascular disease, mechanism, relationship, salt intake, sensitivity

## Abstract

Cardiovascular disease (CVD) is a predominant global health issue, with dietary salt intake recognized as a crucial modifiable risk factor. This review elucidates the multifaceted relationship between salt consumption and CVD, exploring both its direct and indirect effects. While early research emphasized salt's influence on blood pressure, contemporary studies highlight the combined effects of dietary habits and genetic factors on CVD risk. The paper underscores the complex biological mechanisms linking high salt intake to CVD, including its impact on blood pressure, direct cardiovascular effects, immune responses, the role of prostanoids, epigenetic changes, and gut microbiome. Additionally, the review delves into the concept of salt sensitivity and its genetic underpinnings, emphasizing the heightened CVD risk in salt‐sensitive individuals. The potential benefits and challenges of salt substitutes are also discussed. Drawing from various study designs, including epidemiological studies and randomized controlled trials, the review provides a comprehensive understanding of the detrimental effects of excessive salt intake on cardiovascular health, emphasizing the need for refined dietary guidelines and targeted interventions.

**Abbreviations**
CVD: cardiovascular disease
KCL: potassium chloride
RAAS: renin–angiotensin–aldosterone system
RCTs: randomized controlled trials

## 1 Introduction

Cardiovascular disease (CVD) remains a leading cause of mortality worldwide, with lifestyle and dietary factors playing a pivotal role in its onset and progression. Over the past few decades, the medical community's understanding of the relationship between salt intake and CVD has significantly evolved [1]. While earlier studies primarily focused on the direct impact of salt on blood pressure [2], recent research, including the 2023 guideline by the European Society of Hypertension (ESH), has delved into the intricate interplay between dietary habits, genetic factors, and their combined influence on CVD risk [3, 4]. The current epidemic of dietary salt intake highlights a global public health challenge. Excessive salt consumption is prevalent in many countries, significantly exceeding the World Health Organization's recommended daily intake of less than 5 g of salt per day (approximately 2 g of sodium). For instance, in the United States, the average daily salt intake is about 8.5 g, while in China, it averages around 10 g per day. This widespread high salt intake is linked to increased hypertension prevalence and subsequent CVDs [5]. Studies utilizing advanced imaging techniques, such as (23) Na magnetic resonance imaging, have further demonstrated the significant tissue sodium accumulation in individuals with high salt intake, providing deeper insights into the pathophysiological mechanisms underlying hypertension and CVD [6]. Additionally, emerging evidence suggests that high salt intake can alter the gut microbiome, leading to dysbiosis and promoting inflammation, which may further exacerbate cardiovascular risk [7]. Given the ubiquity of salt in modern diets, understanding its impact on CVD is of paramount importance for public health interventions.

The biological mechanisms through which high salt intake may contribute to CVD are complex and multifaceted. They involve both indirect effects through the increase in blood pressure [2] and direct effects on the heart and blood vessels [8]. In addition, excessive salt consumption can have direct detrimental effects on the cardiovascular system, leading to inflammation, oxidative stress, and damage to the heart and blood vessels [9]. Indirectly, high salt intake may influence systems like the renin‐angiotensin system [10], arterial rigidity, and endothelial dysfunction, all integral to hypertension and CVD's onset and progression [11]. Recent research has highlighted the role of epigenetic changes induced by environmental factors, including high salt intake, in CVD. These epigenetic changes can alter gene expression, contributing to the complex interplay between genetics and dietary habits in the development of CVD [12]. The interplay between these direct and indirect mechanisms is complex, warranting further exploration.

It is well‐established that genetic factors influence the connection between salt consumption and CVD [13]. The ESH2023 guideline emphasizes the existence of “salt‐sensitive” individuals who, due to genetic differences, may exhibit heightened sensitivity to salt, leading to significant rises in blood pressure with high salt consumption [3]. Such individuals are potentially at a heightened risk for developing hypertension and CVD [14]. The prevalence and specific genetic variations responsible for this sensitivity, however, remain topics of active research [15]. Recent studies have identified specific genetic polymorphisms, such as those in the SLC4A5 and WNK1 genes, that may contribute to salt sensitivity and hypertension risk [16, 17]. Furthermore, research is ongoing to explore the interactions between these genetic factors and other variables like age, sex, and overall diet [18, 19].

This paper aims to provide a review of the current state of knowledge on the relationship between sodium intake and CVD, highlighting both established findings and areas of contention. Through this, we seek to identify gaps in the literature and provide direction for future research endeavors. Additionally, we aim to explore the implications of recent advancements in genetic and epigenetic research, as well as the role of the gut microbiome, to provide a holistic understanding of sodium intake's impact on cardiovascular health.

## 2 Method

To ensure a comprehensive review, we conducted a narrative literature search using PubMed, Web of Science, and Scopus databases from January 2000 to December 2023. Search terms included combinations of “salt intake,” “sodium,” “CVD,” “hypertension,” “salt sensitivity,” “renin–angiotensin–aldosterone (RAAS),” and “gut microbiome.” We prioritized high‐quality sources such as systematic reviews, meta‐analyses, randomized controlled trials, and key clinical guidelines, including the 2023 ESH Guidelines. Only English‐language publications were included. Relevant reference lists from identified studies were also screened manually to ensure comprehensiveness.

### 2.1 Evidence From Epidemiological Studies and Randomized Clinical Trials

The evidence from various study designs, including cross‐sectional, cohort studies, and randomized controlled trials, underscores the detrimental effects of excessive salt intake on cardiovascular health (Table 1).

**TABLE 1:** The relevant studies linking the relationship between salt intake and CVD risk.

Study	Design	Samples (*n*)	Conclusions
Chowdhury et al. (2016)	Cross‐sectional study	7839	Highest hypertension risk in northwestern (Rangpur) and lowest in eastern (Sylhet) regions, influenced by raw salt intake, poverty, malnutrition, and dietary habits.
Todkar et al. (2009)	Cross‐sectional study	1297	Significant factors for hypertension include age, sex, BMI, additional salt intake, smoking, diabetes, alcohol consumption, and higher socioeconomic status.
Dalton et al. (2011)	Cross‐sectional study	5294	Increasing intervention adherence in high‐risk populations and reinvesting in population‐wide strategies to reduce obesity, smoking, and salt intake may reduce CVD burden cost‐effectively in the UK.
Strazzullo et al. (2009)	Meta‐analysis of cohort studies	177 025	High salt intake is associated with increased risk of stroke and total cardiovascular disease.
He et al. (1999)	Cohort study	9485	High sodium intake is strongly associated with increased risk of CVD and all‐cause mortality in overweight individuals.
Srour et al. (2019)	Cohort study	105 159	Higher consumption of ultra‐processed foods high in sodium is associated with higher risks of cardiovascular, coronary heart, and cerebrovascular diseases.
Cobb et al. (2014)	Methodological advisory	285 530	Methodological issues may account for inconsistent findings in studies relating sodium to CVD.
Cook et al. (2007)	Randomized clinical trial	3126	Sodium reduction, shown to lower blood pressure, may also reduce long‐term cardiovascular event risk.
Sacks et al. (2001)	Randomized clinical trial	412	Reducing sodium intake below 100 mmol/day and following the DASH diet significantly lower blood pressure, with greater effects in combination.
Aburto et al. (2013)	Meta‐analysis of 14 cohort studies and 42 RCTs	5508	Reducing sodium intake lowers blood pressure without negative impacts on blood lipids, catecholamine levels, or renal function, and is linked to a decreased risk of stroke and fatal coronary heart disease.

### 2.2 Cross‐Sectional Study

Excessive salt intake has been consistently linked to an increased risk of hypertension and CVD in the cross‐sectional studies. Studies conducted in diverse settings, including deprived urban areas of Bangladesh [20] and rural Maharashtra [21], have underscored the prevalence of hypertension risk factors, emphasizing the role of high salt consumption. Furthermore, the NHS Health Checks program highlighted the importance of interventions to reduce obesity, smoking, and salt intake as pivotal in mitigating CVD risks [22]. Collectively, these studies underscore the detrimental effects of high salt intake on cardiovascular health and the pressing need for dietary interventions.

### 2.3 Cohort Study

From the viewpoint of cohort studies, a meta‐analysis of prospective studies highlighted a significant association between salt intake, stroke, and overall CVD, underscoring the detrimental effects of high salt consumption on cardiovascular health [9]. A dose‐response meta‐analysis using data from 20 cohort studies found the risk of CVD increased up to 6% for every 1 g increase in dietary sodium intake [23]. Another cohort study focusing on overweight adults revealed that dietary sodium intake was directly correlated with subsequent risk of CVD, emphasizing the importance of sodium regulation in this particular demographic [24]. Additionally, a 2024 large‐scale US prospective cohort study by Gan et al. incorporated both new data from the NIH‐AARP Diet and Health Study (*n* > 400 000) and an updated meta‐analysis. This study found a significant positive association between sodium intake ≥ 2000 mg/day and increased overall and cardiovascular mortality, with a stronger association observed in women (e.g., CVD mortality HR = 1.21 for women in the highest quintile vs. lowest)​. The meta‐analysis component, encompassing over 2 million participants and 80 000 CVD events, supported these findings with a pooled relative risk for CVD events of 1.13 (95% CI: 1.06–1.20) for the highest versus lowest sodium intake categories [25]. Interestingly, a study from the NutriNet‐Santé cohort drew attention to the intake of ultra‐processed foods, which are often high in sodium, and their association with increased CVD risk [26]. While the evidence from cohort studies is compelling, it is essential to consider methodological issues, as highlighted by the American Heart Association, to ensure the robustness of findings and their implications for public health recommendations [27]. However, it is important to acknowledge that not all cohort studies have demonstrated a linear relationship between sodium intake and CVD risk. Some investigations have reported a J‐shaped association, suggesting that both very high and very low levels of sodium intake might be associated with increased cardiovascular events and mortality. For example, findings from the PURE study and other large‐scale cohorts have sparked debates on the optimal range of sodium intake for population health [28, 29, 30]. These inconsistencies highlight the complexity of sodium's role in cardiovascular health and the need for cautious interpretation of observational data, considering factors such as reverse causality, measurement error, and baseline health status.

### 2.4 Evidence From Randomized Clinical Trials

Randomized controlled trials (RCTs) have been instrumental in elucidating the relationship between dietary salt intake and CVD risk. The Trials of Hypertension Prevention (TOHP) series, one of the most comprehensive RCTs on this topic, consistently demonstrated that reduced sodium intake led to significant reductions in blood pressure and CVD risk [31]. Another pivotal RCT, the Dietary Approaches to Stop Hypertension (DASH) study, revealed that a diet low in sodium and rich in fruits, vegetables, and low‐fat dairy products significantly lowered blood pressure, even more than a diet solely focused on sodium reduction [32]. This suggests that while sodium plays a role, other dietary factors also contribute to cardiovascular health. The Systolic Blood Pressure Intervention Trial (SPRINT), which aimed to understand the effects of maintaining lower systolic blood pressure, indirectly emphasized the importance of dietary salt regulation. While the trial was not exclusively focused on salt, the dietary recommendations given to participants included salt reduction, further underscoring its importance in managing blood pressure and, by extension, CVD risk [33]. However, a meta‐analysis of RCTs indicated that while reduced dietary salt was associated with a decrease in blood pressure, its direct effect on cardiovascular events was less clear [34]. This highlights the need for longer‐term RCTs that not only focus on intermediate outcomes like blood pressure but also on hard endpoints like myocardial infarction or stroke. Overall, while RCTs like TOHP, DASH, and SPRINT have provided invaluable insights into the role of salt in cardiovascular health, there remains a need for more extensive, long‐term RCTs that directly assess the impact of salt reduction on CVD outcomes.

### 2.5 Salt Sensitivity and Cardiovascular Implications

Salt sensitivity, characterized by blood pressure variations in response to dietary salt intake, has become a central focus in cardiovascular research [35]. This physiological trait predisposes individuals to pronounced blood pressure fluctuations with alterations in salt consumption. Elevated blood pressure, a consequence of this sensitivity, is a recognized risk factor for CVD [36]. Recent studies indicate that salt‐sensitive individuals, particularly when on a high‐salt diet, face an escalated risk of hypertension and other cardiovascular morbidities [37]. Moreover, certain populations, such as post‐menopausal women, have been found to develop increased salt sensitivity, further emphasizing the need for targeted dietary recommendations [38]. However, the regulation and control of salt sensitivity remain incompletely understood. Several factors contribute to this complex trait, including adipose tissue‐derived cytokines, which play a significant role in modulating blood pressure and inflammation. Renal channels, particularly those involved in regulating salt reabsorption, are critical in maintaining sodium balance and influencing salt sensitivity. Furthermore, epigenetic mechanisms, which involve changes in gene expression without altering the DNA sequence, also contribute to the variability in salt sensitivity observed among different individuals and populations. The genetic architecture of salt sensitivity, notably evident in populations such as the Japanese, underscores the intricate balance between genetics and environmental determinants in cardiovascular risk stratification [39]. Advancements in this domain necessitate a deeper comprehension of the underlying mechanisms of salt sensitivity and their cardiovascular repercussions. Emphasis should be on delineating at‐risk cohorts, optimizing dietary guidelines, and strategizing interventions to counteract the detrimental cardiovascular effects of salt sensitivity.

### 2.6 Salt Substitutes: Implications for Cardiovascular Health and Future Prospects

To mitigate health risks caused by salt, several salt substitutes have been introduced, including potassium chloride (KCl), magnesium salts, and various herbal mixtures. Potassium‐enriched salt substitutes have shown promise in lowering blood pressure, but their direct effects on CVD events remain under‐researched [40]. One study conducted in rural China found that salt substitution was an effective low‐cost strategy for blood pressure control [41]. A randomized controlled trial conducted in China showed that the use of a salt substitute was associated with lower rates of stroke, major cardiovascular events, and all‐cause mortality in individuals aged 60 years or older with a history of stroke or high blood pressure [42]. Another community‐wide study highlighted the potential of salt substitutes to decrease blood pressure and reduce the incidence of hypertension [43]. However, it is crucial to note that while these substitutes can reduce sodium intake, excessive potassium intake can be harmful, especially for individuals with kidney disorders [44]. Recent large‐scale studies, such as the Salt Substitute and Stroke Study (SSaSS), have shown that replacing regular salt with a potassium‐enriched salt substitute can significantly reduce the risk of stroke and other major cardiovascular events, indicating a promising avenue for future public health interventions [42]. As the global community continues to grapple with the health implications of high salt consumption, rigorous clinical trials and population studies will be pivotal in shaping the future landscape of salt substitution and its role in CVD prevention [45].

### 2.7 Mechanisms Linking High Salt Intake to CVD

**FIGURE 1:**
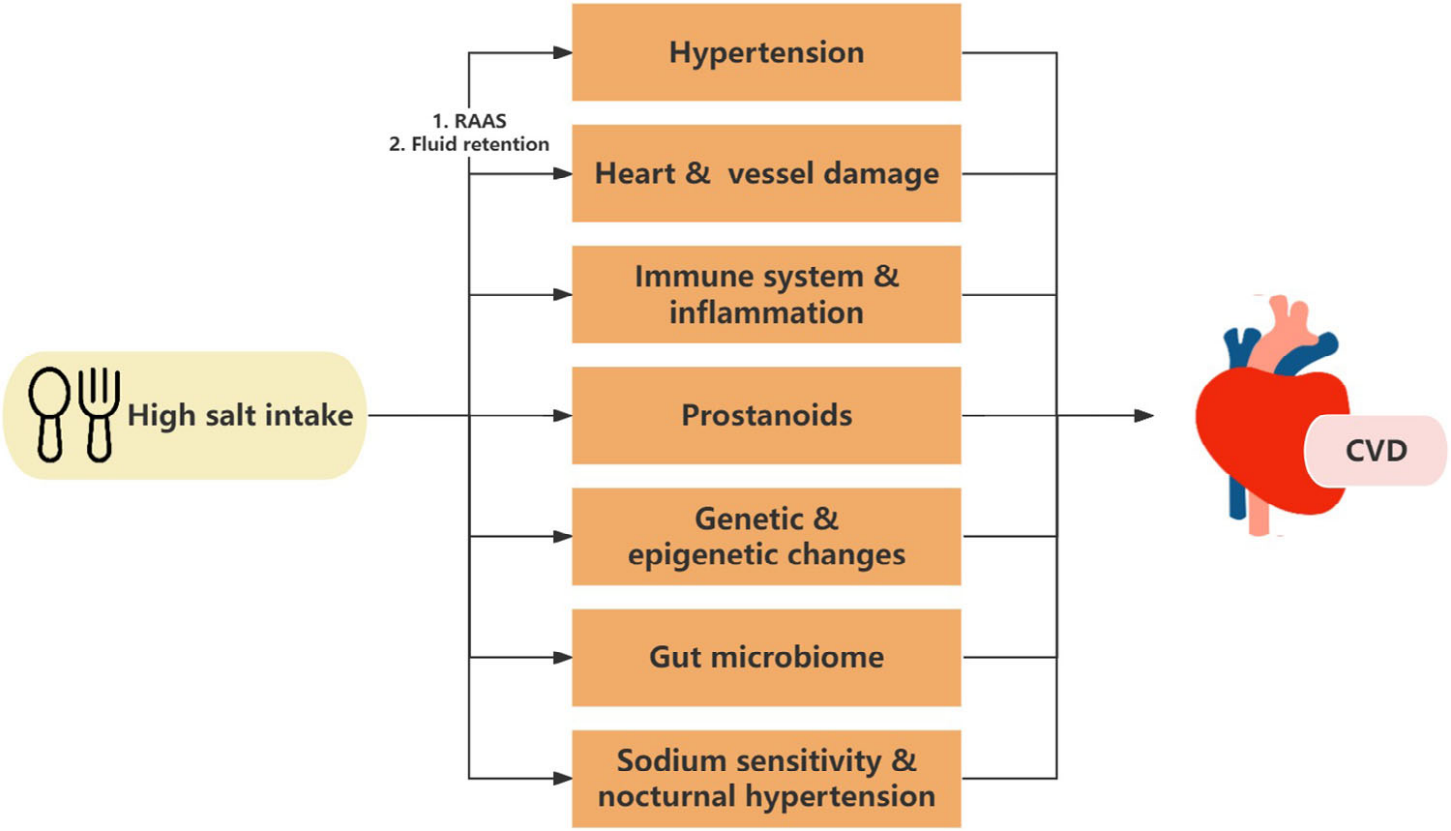
The mechanisms linking high salt intake to CVD.

The mechanisms linking high salt intake to CVD include elevation of blood pressure and hypertension, direct cardiovascular effects, salt‐induced immune responses, and the role of prostanoids in hypertension and CVD (Figure 1).

#### 2.7.1 Elevation of Blood Pressure and Hypertension due to Salt Intake

High salt consumption disrupts the body's fluid balance by promoting fluid retention due to its osmotic properties, leading to increased blood volume, elevated cardiac output, and arterial pressure [46, 47, 48]. This process plays a critical role in the development of nocturnal hypertension, a condition closely associated with CVD. Excessive salt intake can also overstimulate the RAAS, which regulates blood pressure and fluid homeostasis, thereby contributing to sustained hypertension and subsequent cardiovascular damage [49, 50, 51, 52].

Two key studies published in 2000 and 2010 demonstrated that sodium restriction and the use of diuretics can shift the circadian rhythm of blood pressure from a nondipper to a dipper pattern in patients with sodium‐sensitive essential hypertension, underscoring the pivotal role of sodium sensitivity in blood pressure regulation [53, 54]. More recent therapeutic strategies, such as the combination of hydrochlorothiazide and candesartan, have shown promise in managing uncontrolled hypertension and reducing CVD risk [55]. Furthermore, emerging evidence from post‐2020 studies underscores the role of genetic factors in sodium sensitivity and nocturnal hypertension, pointing to new potential targets for intervention [56].

#### 2.7.2 Direct Cardiovascular Effects of Excessive Salt Intake

Beyond its influence on blood pressure, high salt intake can directly harm the heart and blood vessels [57]. For instance, it has been associated with left ventricular hypertrophy, where the heart muscle thickens, potentially leading to heart failure [58]. This condition can reduce the heart's efficiency in circulating blood, leading to various health complications. High salt intake can also cause endothelial dysfunction, impairing blood vessels' dilation ability and contributing to atherosclerosis development [59]. Atherosclerosis involves plaque accumulation inside arteries, narrowing them and obstructing blood flow.

#### 2.7.3 Salt‐Induced Immune and Inflammatory Responses and Cardiovascular Implications

Excessive salt consumption also significantly impacts the immune system [60]. Elevated salt levels initiate an inflammatory reaction, which can lead to atherosclerosis, a primary driver of CVD [61]. This inflammatory process results in the thickening and constriction of blood vessels, diminishing blood circulation, and heightening the likelihood of heart attacks and strokes. Moreover, high‐salt diets foster the creation of pro‐inflammatory Th17 cells [62]. These cells are implicated in hypertension and can disrupt the endothelium, the inner layer of blood vessels [63]. Such endothelial dysfunction is pivotal in the emergence and advancement of CVD, influencing blood vessel dilation and potentially causing blood clot formation.

#### 2.7.4 Role of Prostanoids in Hypertension and CVD

Prostanoids, lipid mediators produced by cyclooxygenase acting on arachidonic acid, play significant roles in both standard physiology and disease states [64, 65]. They are produced extensively in response to various stimuli, including high salt intake, and can contribute to hypertension and CVD. These compounds regulate blood vessel tone, platelet function, and inflammation [66]. Moreover, new insights into the interaction between prostanoids and other signaling molecules highlight potential therapeutic targets for managing hypertension and CVD [67].

#### 2.7.5 Role of Genetic and Epigenetic Changes in Hypertension and CVD

High salt intake contributes to CVD through both genetic and epigenetic mechanisms. Genetic predispositions influence individual responses to salt intake, with certain variants leading to salt‐sensitive hypertension [68]. Epigenetic modifications, such as DNA methylation and histone modifications, also play significant roles. High salt intake is associated with changes in histone methylation, affecting genes involved in inflammation and endothelial function, thereby increasing CVD risk [69, 70]. These epigenetic changes can lead to long‐term cardiovascular damage by maintaining an inflammatory state and endothelial dysfunction.

#### 2.7.6 Role of Gut Microbiome and Epigenetics in Hypertension and CVD

High salt intake has been increasingly recognized to influence CVD through alterations in the gut microbiome. Excessive dietary salt can lead to dysbiosis, characterized by an imbalance in the microbial community within the intestines [71]. This dysbiosis promotes inflammation and hypertension by altering the production of short‐chain fatty acids (SCFAs), reducing beneficial microbiota such as *Lactobacillus*, and enhancing pro‐inflammatory pathways [72]. These changes in the gut microbiome contribute to endothelial dysfunction, a key factor in the development of atherosclerosis and other cardiovascular conditions. Furthermore, high salt intake stimulates the immune system, leading to increased production of pro‐inflammatory cytokines and activation of Th17 cells, which exacerbate vascular inflammation and hypertension [63].

## 3 Summary and Conclusion

CVD remains a global health concern, with dietary salt intake being a significant modifiable risk factor. This review highlights the relationship between salt consumption and CVD. Elevated salt intake influences blood pressure and directly affects the cardiovascular system, leading to conditions like left ventricular hypertrophy and endothelial dysfunction. Genetic predispositions, such as salt sensitivity, can exacerbate these risks. While salt substitutes offer potential benefits, their long‐term implications on CVD are still being studied.

Evidence from various studies underscores the harmful effects of excessive salt intake on cardiovascular health. Recent advancements in genetic and epigenetic research, as well as studies on the gut microbiome, provide new insights into the complex interactions between salt intake and cardiovascular health.

To move forward, it is crucial to refine dietary guidelines, emphasizing salt reduction, and to develop targeted interventions for high‐risk populations. Future research should focus on understanding the underlying mechanisms, identifying at‐risk groups, and exploring potential therapeutic interventions.

## Author Contributions

All authors have read and approved the manuscript. **Fuzhou Han**: writing–original draft preparation. **Wenqiang Li** and Ning Duan: writing–reviewing and editing. Xinlong Hu and **Nan Yao**: investigation, visualization. **Guoyong Yu** and **Jun Qu**: conceptualization, supervision.

## Conflicts of Interest

The authors declare no conflicts of interest.

## Data Availability

The authors have nothing to report.
